# Central Pontine Myelinolysis as a Late Complication After Hyponatremia and COVID-19 Infection

**DOI:** 10.7759/cureus.35191

**Published:** 2023-02-19

**Authors:** Nora I Ivanova, Mihael E Tsalta-Mladenov, Darina K Georgieva, Silva P Andonova

**Affiliations:** 1 Second Clinic of Neurology With ICU and Stroke Unit, University Hospital “Sveta Marina”, Varna, BGR; 2 Department of Neurology and Neuroscience, Medical University “Prof. Paraskev Stoyanov”, Varna, BGR; 3 Second Clinic of Neurology with ICU and Stroke Unit, University Hospital “Sveta Marina”, Varna, BGR; 4 Department of Neurology and Neuroscience, Medical University "Prof. Paraskev Stoyanov", Varna, BGR

**Keywords:** osmotic demyelination syndrome (ods), post-covid, extrapontine myelinolysis (epm), extra pontine myelinolysis (epm), central pontine myelinolysis (cpm), pontine myelinolysis, myelinolysis, covid-19, hyponatremia, osmotic demyelination syndrome

## Abstract

Osmotic demyelination syndrome (ODS) is a rare but serious condition that is hypothesized to be a result of rapid correction of hyponatremia, with a catastrophic prognosis. The foci of demyelination may occur in either the pontine area or within the white matter of the cerebral hemispheres, which denotes a specific clinical presentation. We present the case of a post-COVID-19 patient who was diagnosed with ODS with typical clinical and radiological characteristics of both forms: central pontine myelinolysis and extrapontine myelinolysis. The clinical assessment of ODS encompasses a variety of differential diagnoses, including stroke, neuroinfection, neoplasia, and other demyelinating diseases. A specific characteristic of ODS is the delayed clinical manifestation after the hyponatremic state. Furthermore, it is noteworthy that there might be discrepancies between the clinical manifestations and the neuroimaging findings. The association between COVID-19 and ODS is unclear at the moment, although it can possibly be explained by the viral infection through multiple mechanisms such as renal dysfunction, diarrhea, or vomiting. ODS should be considered in cases of hyponatremia and neurological deterioration during the course of COVID-19 infection. Despite the fact that early detection and treatment of this syndrome can reduce the risk of short-term mortality and long-term disability, they do not guarantee complete recovery.

## Introduction

Osmotic demyelination syndrome (ODS) is a severe, uncommon complication that is considered to be caused by rapid correction or overcorrection of hyponatremia, although the exact pathogenesis remains unknown [1]. Hyponatremia is defined as serum sodium below 135 mmol/L [2]. It usually presents with central pontine myelinolysis (CPM), in which the focus of demyelination is in the pontine region. Another form of ODS is extrapontine myelinolysis (EPM), in which demyelination occurs in the white matter of the cerebral hemispheres [3]. CPM was first described by Adams in 1959 based on autopsy findings of symmetric pontine myelinolysis in patients with alcoholism and malnutrition [4]. ODS has typically been described in alcoholics, liver transplant recipients, and patients with rapid osmolar shifts. Some other predisposing factors have been reported, such as hypokalemia, hypophosphatemia, diabetes mellitus, anorexia nervosa, hyperemesis gravidarum, and severe burns [5]. We report the case of a post-COVID-19 patient with ODS and typical neuroimaging findings suggestive of CPM and EPM.

## Case presentation

A 55-year-old woman presented with complaints of 10 days of unintentional and uncontrolled rhythmic movements of the four limbs, which she described as “twitching.” On the current admission, the neurological examination revealed hyporeflexia for the deep tendon reflexes of all limbs and oral automatisms.

The blood samples revealed that the serum electrolytes were slightly decreased, whereas the rest of the examined parameters were within the reference range (Table 1).

**Table 1:** Laboratory parameters of the patient during the different time points of the follow-up. H: higher than the reference range; L: lower than the reference range

	On the current admission due to CMS	Three days after the admission due to CMS	One week after the admission due to CMS	On the previous admission due to COVID-19	Two days after the admission due to COVID-19	Reference range of the examined laboratory values
White blood cell count (10^9^/L)	9.46	5.03	7.52	5.10	3.7	3.60–10.50
Neutrophil count (10^9^/L)	7.19	3.69	6.30	3.81	2.09	1.50–7.70
Red blood cell count (10^12^/L)	4.31	3.99	3.68 (L)	5.13	4.9	4.00–5.65
Hemoglobin (g/L)	128	121 (L)	110 (L)	152	131	125–172
Platelet count (10^9^/L)	203	179	190	149	149	140–440
Glucose (mmol/L)	6.4 (H)	6.7 (H)	5.9	7.3 (H)	6.6 (H)	4.1–5.9
Serum creatinine (µmol/L)	48 (L)	63	66	64	53 (L)	62–115
Uric acid (mmol/L)	1.0 (L)	4.1	3.8	4.4	4.8	3.2–8.2
Aspartate transaminase (U/L)	18.7	41.4	38.5 (H)	290 (H)	144.2 (H)	0.0–34
Alanine transaminase (U/L)	18.9	24.7	28.1	84 (H)	63.2 (H)	10–49
High-sensitivity C-reactive protein (mg/L)	26.17 (H)	29.55 (H)	13.14 (H)	14.02 (H)	8.52 (H)	0–5.0
Sodium (mmol/L)	130 (L)	138	136	116 (L)	133	132–146
Potassium (mmol/L)	2.4 (L)	3.9	4.5	2.9 (L)	3.3 (L)	3.5–5.5
Chloride (mmol/L)	93 (L)	101	104	72 (L)	99	99–109
International normalized ratio	1.03	1.22 (H)	1.21 (H)	0.92	1.01	0.9–1.15
Prothrombin time (seconds)	14.0	15.3	15.1 (H)	13.9	16.1	11.5–14.8
Activated partial thromboplastin time (seconds)	39.3 (H)	31.4	32.4	41.7 (H)	36.5	25.4–36.8

A computed tomography (CT) of the head was normal, and because of the discrepancy between the neurological condition and the neuroimaging finding, the patient underwent a magnetic resonance imaging (MRI) of the head with contrast, again without any significant changes except for an incidental parietal subcutaneous hemangioma. The patient was treated for a suspected ischemic stroke with negative MRI visualization with aspirin 325 mg orally once a day, along with the correction of the hypoosmolar state.

**Figure 1:**
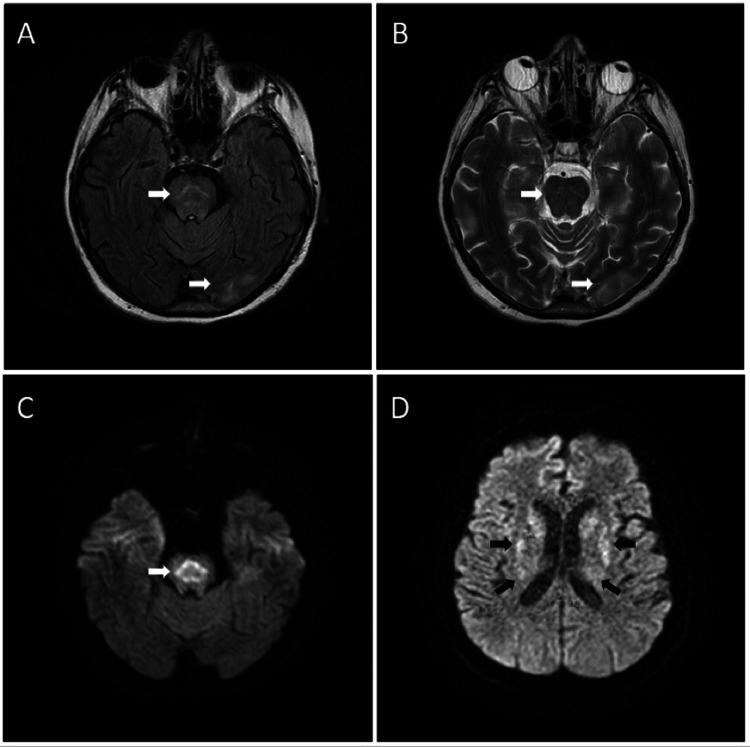
Follow-up MRI imaging after seven days. A: Axial T2 sequence and B: Axial T2/fluid-attenuated inversion recovery - High signal intensity zone in the central pontine region and in the temporooccipital subcortical white matter. C and D: Axial diffusion-weighted imaging - High signal intensity zone in the central pontine region with a typical “trident” or “piglet” sign and in the basal ganglia, including the putamen, nucleus caudatus, and the lateral part of the thalamus bilaterally. Arrows: Pointing the major pathological findings - hyperintense lesions specific for osmotic demyelination syndrome with pontine and extrapontine localization.

Until this point (48 hours after admission), we observed a severe deterioration in neurological status manifested by bulbar syndrome, namely, dysarthria, dysphagia, spastic quadriplegia, and resting tremor in all limbs. She was moved to the neurological intensive care unit for further investigations and treatment. At this point, the blood samples revealed no abnormalities (Table 1). A lumbar puncture was performed, and the cerebrospinal fluid analysis showed no abnormalities. Due to the progression of the neurological deficit, a second MRI of the head was performed one week after the severe neurological deterioration. It revealed a pathologic high signal intensity zone on T2-weighted images in the central pontine region and in the basal ganglia-putamen, nucleus caudatus, and the lateral part of the thalamus bilaterally. There was a correspondingly low signal on T1-weighted images and restricted diffusion on diffusion-weighted images. Additionally, a small hyperintense zone was visualized in temporooccipital white matter subcortically on T2 images (Figure 1). The described brain changes were highly indicative of ODS, with both pontine and extrapontine osmolar myelinolysis present.

Because of the extrapyramidal syndrome, amantadine sulfate was included in the therapy but without any significant improvement. During the follow-up, the patient had polyuria with about 6 L daily and normal serum glucose levels, and, therefore, diabetes mellitus was ruled out. Liver enzymes were slightly elevated, which referred to reactive hepatitis due to a previous antibiotic intake.

In the present case, we performed various laboratory tests (blood samples, hemocultures, urine, and cerebrospinal fluid analysis) and neuroimaging examinations (CT of the head and MRI with contrast) to rule out other differential diagnoses, such as acute stroke, neuroinfection, neoplasm, or other demyelinating diseases. Two lumbar punctures showed no abnormalities, and no inflammatory or neoplastic cells were detected. Viral markers for *Borrelia burgdorferi*, HIV, syphilis, herpes simplex, herpes zoster, and enteroviruses were also negative. Due to the typical course of the disease and the clinical and neuroimaging findings, a diagnosis of ODS with both pontine and extrapontine myelinolysis was made.

After improving the hemodynamics and the dyselectrolytemia, the patient was discharged from the hospital with a diagnosis of ODS in a chronic condition with severe quadriparesis, rigidity in all four limbs, dysarthria, and dysphagia.

The previous medical history revealed that 14 days prior to the current hospital stay, the patient was discharged from another clinic where she was treated for a moderately severe COVID-19 infection. She was admitted there because of a five-day history of fatigue, muscle aches, subfebrile temperature, diarrhea, and vomiting. On admission, the nasopharyngeal swab and subsequent polymerase chain reaction test for SARS-COV-2 (COVID-19) were positive, and the chest X-ray revealed pneumonia. The blood samples revealed increased C-reactive protein, hyperferritinemia, elevated liver enzymes, and a severe hypoosmolar state (Table 1). She received isotonic (0.9%) saline solution for the next two days, resulting in a rapid normalization of the sodium levels (Table 1).

## Discussion

The most common cause of ODS is hyponatremia, with subsequent osmotic stress affecting mainly the oligodendrocytes that regulate tissue osmotic pressure [4]. In response to hyponatremia, water flows into brain cells. They adapt to this condition by reducing the intracellular tonicity through the transport of intracellular solutes to the extracellular compartment. In this way, the brain cells remove the excess water to prevent cerebral edema [6].

A COVID-19 viral infection can also be considered a risk factor as it causes hyponatremia through different mechanisms, namely, poor food intake, vomiting, diarrhea, and kidney injury [7]. The rapid correction of sodium levels with more than 0.5 mmol/L/hour and exposure to high osmolarity levels leads to hypernatremia, decompensation, and dehydration of white matter, resulting in apoptosis of the pontine cells and demyelination of the neuronal fibers due to the inability of the brain to adapt to the new equilibrium [8].

Hyponatremia is classified as acute or chronic according to the time of onset. In the first case, it occurs within 48 hours and the clinical manifestations are usually more severe. According to the current clinical practice guidelines on the diagnosis and treatment of hyponatremia, the correction of sodium levels should be around 10 mmol/L per day for both acute and chronic hyponatremia. When ODS is expected in case of hypokalemia, alcoholism, liver disease, or malnutrition, a lower limit of 8 mmol/L is recommended [2]. Hyponatremia of less than 114 mmol/L in conjunction with hypokalemia is a poor prognostic factor with a notable reduction in vigilance and a higher lethal rate [9].

The description of other cases of patients with COVID-19 infection combined with ODS implies a connection between the two states and a possibility of considering the viral infection as a risk factor for the onset or progression of ODS [6].

Intravital confirmation of diagnosis became possible with the introduction of CT and MRI into neurological diagnostics [10]. The imaging method of choice is MRI, which is very sensitive for discovering myelinolysis even in mild or asymptomatic cases [9]. Typical findings on MRI in patients with CPM and EPM are hyperintense lesions of the pons and extrapontine structures on T2-weighted sequences with corresponding hyperintensity on T1-weighted sequences [9]. The MRI of the brain may initially be normal, and MRI findings do not necessarily correlate with the clinical presentation [11]. In our patient, the first MRI scan showed no abnormalities as the signs of myelinolysis were detected nearly two weeks from the onset of the symptoms.

The clinical manifestation of myelinolysis is frequently delayed for a period ranging from one to 14 days after a brief, symptom-free, or stable clinical interval; there is a secondary deterioration in the neurological deficit induced by ODS [5]. The opposite scenario is also possible; patients can have typical findings of CPM on an MRI brain scan without associated clinical findings [11]. In the present case, there was severe hyponatremia about two weeks prior to the severe neurological deterioration, which is again indicative of ODS.

The demyelinating focus is different in CPM and EPM, as these conditions can be both present. In CPM, it occurs in the pons, and in EPM, lesions are typically bilateral and symmetrical and involve the thalamus, cerebellum, nucleus caudatus, nucleus lentiformis, putamen, hippocampus, and cortex [12]. CPM presents clinically with dysarthria, dysphagia, and flaccid quadriparesis, which then turns into spastic or locked-in syndrome. In some cases, neuropsychiatric changes may be present. If the tegmentum is involved, oculomotor abnormalities occur. Clinical manifestations of EPM include tremors, ataxia, dystonia, rigidity, mutism, and parkinsonism [5].

The most common MRI findings in ODS are defined as hyperintensities or lesions with an increased brightness on T2-weighted imaging [12]. Brainstem lesions can be classified as focal or diffuse. In differential diagnosis, they should be distinguished from tumors, infections, and processes secondary to systemic conditions, such as ODS, vasculitis, traumatic injuries, degenerative disorders, and ischemic pathology. MRI is the most suitable method of evaluating ODS, but, nevertheless, the findings are not disease-specific [13].

The definite diagnosis of ODS should be based on the neuroimaging findings, the typical constellation for a hypoosmotic state from the blood sample analysis, and a correlation to the patient’s history and clinical findings.

The prognosis for ODS is severe. Usually, patients with ODS require prolonged neurorehabilitation. Often, symptoms are irreversible or partially reversible. The mortality rate is about 30%, following a significant decrease in recent years. Another 30% of patients will have severe disabilities and will need help with their daily living activities [14].

## Conclusions

ODS is a rare condition associated with various risk factors and unfavorable clinical outcomes. The presence of hyponatremia and neurological deterioration during COVID-19 infection requires consideration of ODS. Even though early detection and treatment of this syndrome can reduce the risk of short-term mortality and long-term disability, they do not guarantee complete recovery.
